# Associations of Physical Function and Physical Activity With COVID-19-Like Symptoms

**DOI:** 10.1093/geroni/igab046.2080

**Published:** 2021-12-17

**Authors:** Marguerita Saadeh, Amaia Calderón-Larrañaga, Davide Vetrano, Philip Von Rosen, Anna-Karin Welmer

**Affiliations:** 1 Aging Research Center, Karolinska Institutet, Stockholm, Stockholms Lan, Sweden; 2 Karolinska Institutet, Solna, Stockholms Lan, Sweden; 3 Physiotherapy, Stockholm, Stockholms Lan, Sweden; 4 Karolinska Institutet 17175, Stockholms Lan, Sweden

## Abstract

Physical function and physical activity have been associated with health outcomes related to the cardiopulmonary and immune systems, but the extent to which they are related to the risk of developing COVID-19-like symptoms remains unclear. We aimed to explore these associations among Swedish older adults. We analyzed data from 904 individuals aged ≥68 years from the population-based Swedish National study on Aging and Care in Kungsholmen. COVID-19-like symptoms were assessed by phone interview (March-June 2020) and included fever, cough, sore throat and/or a cold, headache, pain in muscles, legs and joints, loss of taste and/or odour, breathing difficulties, chest pain, gastrointestinal symptoms and eye inflammation. Muscle strength, mobility, and physical activity were objectively examined in 2016-2018. Data were analyzed using logistic regression models and stratifying by age. During the first outbreak of the pandemic, 325 (36%) individuals from our sample developed COVID-19-like symptoms. Those with longer time to perform the chair stand test had an odds ratio (OR) of 1.5 (95% confidence interval [CI] 1.1-2.1) for presenting with COVID-19-like symptoms compared to those with a faster time to perform the test, after adjusting for potential confounders. The risk was even higher among people aged ≥80 years (OR: 2.6; 95% CI 1.5-4.7). No significant associations were found for walking speed or moderate-to-vigorous physical activity. A weaker muscle strength, especially among the oldest-old adults, may contribute to higher odds of developing COVID-19-like symptoms, emphasizing the need to maintain sufficient levels of muscle strength in old age.

